# Zero-Delay Joint Source Channel Coding for a Bivariate Gaussian Source over the Broadcast Channel with One-Bit ADC Front Ends

**DOI:** 10.3390/e23121679

**Published:** 2021-12-14

**Authors:** Weijie Zhao, Xuechen Chen

**Affiliations:** 1 School of Computer Science and Engineering, Central South University, Changsha 410083, China; zhaowj9090@gmail.com; 2 School of Electronics and Information Technology, Sun Yat-sen University, Guangzhou 510275, China

**Keywords:** joint source channel coding, zero-delay transmission, broadcast channel, bivariate Gaussian sources, average distortion, one-bit ADC

## Abstract

In this work, we consider the zero-delay transmission of bivariate Gaussian sources over a Gaussian broadcast channel with one-bit analog-to-digital converter (ADC) front ends. An outer bound on the conditional distortion region is derived. Focusing on the minimization of the average distortion, two types of methods are proposed to design nonparametric mappings. The first one is based on the joint optimization between the encoder and decoder with the use of an iterative algorithm. In the second method, we derive the necessary conditions to develop the optimal encoder numerically. Using these necessary conditions, an algorithm based on gradient descent search is designed. Subsequently, the characteristics of the optimized encoding mapping structure are discussed, and inspired by which, several parametric mappings are proposed. Numerical results show that the proposed parametric mappings outperform the uncoded scheme and previous parametric mappings for broadcast channels with infinite resolution ADC front ends. The nonparametric mappings succeed in outperforming the parametric mappings. The causes for the differences between the performances of two nonparametric mappings are analyzed. The average distortions of the parametric and nonparametric mappings proposed here are close to the bound for the cases with one-bit ADC front ends in low channel signal-to-noise ratio regions.

## 1. Introduction

Traditional digital communication systems, based on Shannon’s separation principle between source and channel coding [1], concentrate on mappings with long block lengths. Although these separated systems are not very robust to the channel variation, optimality can be achieved given that no constraints are considered in terms of complexity and delay. However, these systems have become unsuitable for certain emerging applications that require transmission in extreme latency constraints, such as those involving the internet of things (IoT) technologies [2] or wireless sensor networks (WSNs) [3]. Based on these applications scenarios, strict delay constraints are present owing to the near real-time monitoring and feedback between users and the underlying physical systems. For example, with the full realization of the industry 4.0 revolution in the forthcoming sixth-generation (6G) connection standards, machine controls are expected to achieve real-time operations with guaranteed microsecond delay jitter [4].

As a result, we consider the extreme case of JSCC, zero-delay transmission, where a single source sample is transmitted over a single use of the channel.

A well-known approach for zero-delay transmission is the linear scheme, in other words, the uncoded scheme that can achieve the minimum squared distortion for a Gaussian source transmitted over an additive white Gaussian noise (AWGN) channel with an input power constraint [5]. In the point-to-point setting, the linear scheme is an alternative to the optimal separate source and channel coding (SSCC). The linear scheme outperforms SSCC in terms of simplicity and zero-delay, specifically in applications that include the uncoded video transmission [6] and real-time control system for IoT [7]. However, at times, the linear scheme is not sufficient for exploiting the additional degrees-of-freedom available in the multi-terminal system. In many multi-terminal scenarios, both the SSCC and linear schemes underperform in terms of optimality. In [8], Bross et al. proved that, for the transmission of a memoryless bivariate Gaussian source over the Gaussian broadcast channel (GBC), the uncoded scheme achieves the optimality whenever the channel signal-to-noise ratio (CSNR) is below a certain threshold. To date, various zero-delay analog mappings, including parametric and nonparametric mappings, have been proposed for different scenarios [9,10,11]. In [12,13,14], hybrid digital and analog (HDA) schemes for zero-delay transmission to obtain superior performances to the uncoded schemes in various multi-terminal cases have been reported.

The analog-to-digital converter (ADC) plays an important role in the receiving antenna as the key component of the front end of the digital receiver. The power consumption of the ADCs increases exponentially with its resolution [15]. The above drawback leads to a growing concern of the energy consumption of the receiving ends. In [16], Jeon et al. proposed computationally efficient yet near-optimal soft-output detection methods for coded millimeter-wave (mmWave) multiple input multiple output (MIMO) systems with low-precision ADCs. The proposed method provides significant gains compared to the existing techniques in the same setting with the use of low-precision ADCs. In [17], Dong et al. analyzed the uplink performance of a multiuser massive MIMO system with spatially correlated channels with low-precision ADCs. Herein, we consider an extreme case, namely, one-bit ADCs, which can be realized by a simple threshold comparator, regardless of the need for mechanical gain control [18,19].

The advantages of the one-bit ADC front end on the performance of a specific communication system have been analyzed in the literature for numerous models. In [20], a low-complexity, near-maximum-likelihood-detection (near-MLD) algorithm was presented for an uplink massive MIMO system with one-bit ADCs, where the authors prove that the proposed algorithm achieves near-MLD performance, while the computational complexity was reduced compared with the existing method. In [21], supervised-learning technique in machine learning is exploited to provide efficient and robust channel estimation and data detection in massive MIMO systems with one-bit ADCs. In [22], conditional adversarial networks in the channel estimation for a massive MIMO system with one-bit ADCs were studied. Channel estimation algorithms were developed to exploit the low-rank property of mmWave channels with one-bit ADCs at the receivers [23]. The proposed methods achieve better channel reconstruction than compressed sensing-based techniques aiming at exploitation of sparsity of mmWave channels. In [24], Morteza et al. considered the zero-delay transmission of a Gaussian source over an AWGN channel with one-bit ADC front end and correlated side information at the receiver. Numerical results demonstrate the periodicity of the optimized encoder mapping.

Information transmission over broadcast channels is an appealing problem in multi-terminal communications. Numerous efforts have been expended in recent studies that focused on low/zero-delay transmission in this case. The asymptotic energy-distortion performance of zero-delay communication was investigated in [25] under the setting of Gaussian broadcasting. A constant lower bound on the energy-distortion dispersion pair is derived as well. In [26], the authors focused on the optimization of parametric continuous mappings that satisfy the individual quality of service requirements. By contrast [27], Tian et al. provided a complete characterization of the achievable distortion region for the above problem. In [28], Hassanin et al. proposed a low complexity, low delay, analog JSCC system based on the extensions of nested quantization techniques. In [29], they further presented the procedure for optimization of the decoding functions and analyzed the assessed performance improvements. For the case of lossy transmission of a Gaussian source over a GBC in instances where there is correlated side information at the receiver, a practical, low delay digital scheme was studied [30]. With the idea of layered superposition transmission and the successive canceling method, the proposed scheme shows higher accuracy of source reconstruction compared with SSCC. In [31], Saleh et al. studied the tradeoff between the distortion of the sources and the error of the interference estimation subject to the setting of the joint recovery of a bivariate Gaussian source and interference over the two-user Gaussian degraded broadcast channel in the presence of a common interference.

In this work, considering extremely low delay and low energy consumption requirements, we focus on the zero-delay JSCC communications system for a bivariate Gaussian source over a bandwidth-matched Gaussian broadcast channel with two receivers. Both of the receivers are equipped with a one-bit ADC front end. To the best of our knowledge, there are few works that have investigated this scenario. The main contributions of this work are summarized as follows:

- Under mean squared error (MSE) distortion criterion, an outer bound on the conditional distortion region is derived.
- Two types of nonparametric mappings are proposed. The first one is based on the joint optimization between the encoder and decoder under an iterative algorithm. In the second method, the implicit functions for the optimal encoder and decoder are derived. Employing the necessary condition mentioned above, the optimized encoder was obtained using the gradient descent method. To the best of our knowledge, there is no previous work that derives the necessary condition of the optimal encoder for the transmission of correlated Gaussian sources over the broadcast channel with one-bit ADC front ends. Hence, our contribution lies in obtaining an encoder mapping that satisfies the necessary derived condition numerically and reveals its structure in three-dimensional space.
- Examining the optimized encoder obtained and imitating the property of its structure, we propose a series of parametric function curves applied to the system model. These mappings are easy to implement.

The remainder of the paper is organized as follows. In Section 2, we introduce the system model and explain the problem of interest. Section 3 focuses on the theoretical bounds under the setting of infinite resolution ADC and one-bit ADC front end. In Section 4, the analysis of the necessary conditions of the optimal encoder and decoder for the proposed system model is presented, and the optimized encoder mappings obtained via the aforementioned necessary condition with the use of two different algorithms are discussed. In Section 5, several new parametric mapping structures are presented. In Section 6, numerical results and analyses are provided and Section 7 concludes the paper.

Notation: Throughout the paper, the uppercase and lowercase letters denote random variables and their realizations, respectively. p(·) and Pr(·) represent the probability density function (pdf) and probability, respectively. The standard normal distribution and its pdf are denoted by N(0,1) and ϕ(·), respectively. Q(·) denotes the complementary cumulative distribution function of the standard normal distribution.

## 2. Problem Formulation

**Figure 1:**
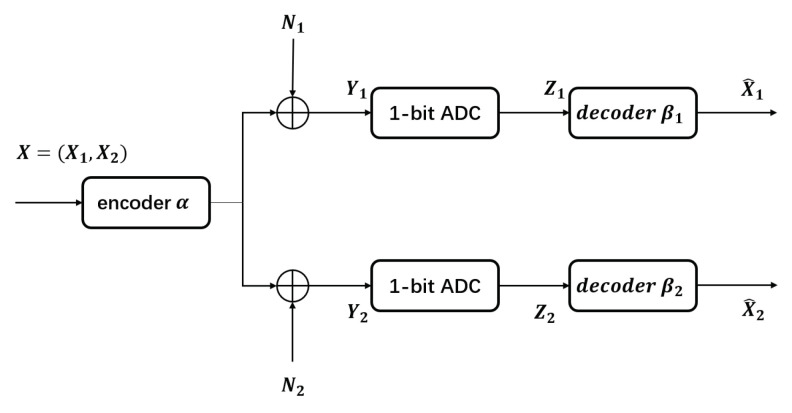
Broadcasting bivariate Gaussian sources with one-bit ADC front end.

We consider the transmission of correlated Gaussian sources over Gaussian broadcast channels with one-bit ADC at the receivers. The setup is illustrated in Figure 1. Herein $X=(X_{1},X_{2})$ denotes a couple of memoryless and stationary bivariate Gaussian sources with zero mean and variance $\sigma_{X}^{2}$. The covariance matrix of the two sources is presented below,

(1) $$\begin{array}{c} \left[\begin{array}{cc} \sigma_{X}^{2} & \rho \sigma_{X}^{2}\\ \rho \sigma_{X}^{2} & \sigma_{X}^{2} \end{array}\right], \end{array}$$

where ρ∈[0,1]. The source vector X is transformed into a one-dimensional channel input *V* with the use of a nonlinear mapping function $V=\alpha (X_{1},X_{2})$. The Gaussian memoryless broadcast channel is given by    

(2) $$\begin{array}{c} Y_{i}=\alpha \left(X_{1},X_{2}\right)+N_{i},\;i=1,2, \end{array}$$

as shown in Figure 1, where $Y_{i}$ is the channel output for channel *i*, and $N_{i}$ is the AWGN, independent of $X_{1}$ and $X_{2}$ for channel *i*, with zero mean and variance $\sigma_{n_{i}}^{2}$. Without loss of generality, we assume $\sigma_{n_{1}}^{2}<\sigma_{n_{2}}^{2}$. At the *i*-th receiver, the noisy signal $Y_{i}$ is quantized with a one-bit ADC, Γ(.). The output of the ADC is

(3) $$\begin{array}{c} Z_{i}=\Gamma \left(Y_{i}\right)=\left\{\begin{array}{cc} 0\; & Y_{i}\geq 0\\ 1\; & Y_{i}<0. \end{array}\right. \end{array}$$

The decoder observing the ADC output reconstructs the source $X_{i}$ as ${\hat{X}}_{i}=\beta_{i}\left(Z_{i}\right)$ where $\beta_{i}\left(\cdot \right)$ denotes the *i*-th decoder.

In this paper, we assume that the encoding mapping α follows an average power constraint,

(4) $$\begin{array}{c} E[\|\alpha \left(X_{1},X_{2}\right){\|}^{2}]\leq P. \end{array}$$

The average MSE distortion measure is used, which is given by

(5) $$\begin{array}{cc} \bar{D} & =\bar{MSE}=\frac{1}{2}\sum_{i=1}^{2}D_{i}=\frac{1}{2}\sum_{i=1}^{2}E\left[{\left(X_{i}-{\hat{X}}_{i}\right)}^{2}\right]. \end{array}$$

Our target is to find the optimal source mapping function α and the decoding function $\beta_{i}$ to minimize the average MSE in (5) subject to the average power constraint in (4).

## 3. Preliminaries

### 3.1. The Average Distortion Bound When Infinite Resolution ADC Front Ends Are Adopted

In [27], the authors derived the characterization of the achievable distortion region $D(\sigma_{X}^{2},\rho ,P,\sigma_{n_{1}}^{2},\sigma_{n_{2}}^{2})$. The minimum and maximum values of $D_{1}$ are deduced as follows,

(6) $$\begin{array}{c} D_{1}^{min}=\frac{\sigma_{n_{1}}^{2}\sigma_{X}^{2}}{P+\sigma_{n_{1}}^{2}},\;D_{1}^{max}=\sigma_{X}^{2}\frac{\left(1-\rho^{2}\right)P+\sigma_{n_{1}}^{2}}{P+\sigma_{n_{1}}^{2}}. \end{array}$$

Then, for each $D_{1}\in \left[D_{1}^{min},D_{1}^{max}\right]$,

(7) $$\begin{array}{c} D_{2}\left(P,D_{1},\sigma_{X}^{2},\rho ,\sigma_{n_{1}}^{2},\sigma_{n_{2}}^{2}\right)=\min_{\left(D_{1},d_{2}\right)\in D\left(\sigma_{X}^{2},\rho ,P,\sigma_{n_{1}}^{2},\sigma_{n_{2}}^{2}\right)}d_{2}. \end{array}$$

Subsequently, the average distortion can be obtained by $\bar{D}=1/2\left(D_{1}+D_{2}\right)$ for this distortion pair $\left(D_{1},D_{2}\right)$. We select the smallest average distortion ${\bar{D}}_{\min}$ as the bound of average distortion for the setting of bivariate Gaussian sources over the broadcast channel with infinite resolution ADC front ends.

### 3.2. The Average Distortion Bound When One-Bit ADC Front Ends Are Adopted

The genie-aided distortion region for the transmission of correlated Gaussian sources over a GBC with one-bit ADC front ends, $D_{c}^{ADC}\left(\sigma_{X}^{2},\rho ,P,\sigma_{n_{1}}^{2},\sigma_{n_{2}}^{2}\right)$, consists of all pairs of $\left(D_{1|2}^{ADC},D_{2}^{ADC}\right)$ such that

(8) $$\begin{array}{cc} D_{1|2}^{ADC} & \geq \frac{\sigma_{X}^{2}\left(1-\rho^{2}\right)}{2^{2\left(h\left(Q\left(\sqrt{\gamma P/\sigma_{n_{1}}^{2}}\right)\right)-h\left(Q\left(\sqrt{P/\sigma_{n_{1}}^{2}}\right)\right)\right)}},\;D_{2}^{ADC}\geq \frac{\sigma_{X}^{2}}{2^{2\left(1-h\left(Q\left(\sqrt{\gamma P/\sigma_{n_{2}}^{2}}\right)\right)\right)}}, \end{array}$$

for some γ∈[0,1]. A proof of (8) is given in Appendix A.

In the same way as in Section 3.1, we can obtain the average distortion bound.

## 4. Nonparametric Mappings

In this section, we proceed to develop two types of nonparametric mappings using the Lagrange multiplier method. We are going to study the optimal mapping such that the average distortion is minimized subject to the average power constraint.

Using the Lagrange multiplier method, we turn the constrained optimization problem of minimizing (5) subject to (4) into an unconstrained problem by forming the Lagrange cost function,

(9) $$\begin{array}{c} J\left(\alpha ,\beta_{1},\beta_{2}\right)=\sum_{i=1}^{2}\frac{1}{2}E\left[{\left(X_{i}-{\hat{X}}_{i}\right)}^{2}\right]+\lambda E\left[\|\alpha \left(X_{1},X_{2}\right){\|}^{2}\right]. \end{array}$$

Therefore, our target turns into minimizing the unconstrained problem as

(10) $$\begin{array}{c} \min_{\alpha ,\beta_{1},\beta_{2}}J\left(\alpha ,\beta_{1},\beta_{2}\right). \end{array}$$

For a given λ, if the solution of the unconstrained problem (10) satisfies the average power constraint in (4), it is proven that the above solution also solves the constrained problem [32].

Herein, ${\mathrm{MSE}}_{i}$ is expressed as

(11) $$\begin{array}{cc} {\mathrm{MSE}}_{i}= & \int \int Pr\left(Z_{i}=0|X_{1}=x_{1},X_{2}=x_{2}\right)\times p\left(x_{1},x_{2}\right)\times {\left(x_{i}-\beta_{i}\left(0\right)\right)}^{2}dx_{2}dx_{1}\\ & +\;\int \int Pr\left(Z_{i}=1|X_{1}=x_{1},X_{2}=x_{2}\right)\times p\left(x_{1},x_{2}\right)\times {\left(x_{i}-\beta_{i}\left(1\right)\right)}^{2}dx_{2}dx_{1}. \end{array}$$

The actual transmission power is expressed as

(12) $$\begin{array}{c} P_{act}=\int \int p\left(x_{1},x_{2}\right)\alpha \left(x_{1},x_{2}\right)dx_{2}dx_{1}. \end{array}$$

### 4.1. Nonparametric Mapping I

Herein, we proceed in a way similar to the vector quantizer design [33] by formulating the necessary conditions for optimality with the use of the discretization operation. This scheme is based on joint optimization with iteration between the mappings at the transmitter and receiver.

Note that the minimization of (10) is still difficult to achieve owing to the interdependencies between the components to be optimized. Therefore, we bypass this problem by optimizing the problem iteratively, one component at a time, while we keep the other components fixed.

Assuming that the decoders $\left(\beta_{1},\beta_{2}\right)$ are fixed, the optimal encoding mapping α can be expressed as   

(13) $$\begin{array}{c} \alpha =\underset{\alpha }{\arg\min}\left\{\sum_{i=1}^{2}\frac{1}{2}E\left[{\left(X_{i}-{\hat{X}}_{i}\right)}^{2}\right]+\lambda E\left[\|\alpha \left(X_{1},X_{2}\right){\|}^{2}\right]\right\}. \end{array}$$

Note that if the joint pdf $p(x_{1},x_{2})$ in (12) is nonnegative, the optimization of (13) can be modified in the following way,

(14) $$\begin{array}{c} \alpha \left(x_{1},x_{2}\right)=\underset{v\in R}{\arg\min}\left\{\frac{1}{2}\sum_{i=1}^{2}{\mathrm{MSE}}_{i}+\lambda v^{2}\right\}. \end{array}$$

Assuming that the encoder α is fixed, the optimal decoder is the minimum MSE (MMSE) estimator of $X_{i}$ given $Z_{i}$. The MMSE estimation for user *i* is given by

(15) $$\begin{array}{cc} {\hat{x}}_{i} & =\beta_{i}\left(z_{i}\right)=E\left[x_{i}|z_{i}\right]=\int x_{i}p\left(X_{i}=x_{i}|Z_{i}=z_{i}\right)dx_{i}\\ \; & =\frac{\int \int x_{i}p\left(x_{1}\right)p\left(x_{2}|x_{1}\right)Pr\left(Z_{i}=z_{i}|V=\alpha \left(x_{1},x_{2}\right)\right)dx_{2}dx_{1}}{\int \int p\left(x_{1}\right)p\left(x_{2}|x_{1}\right)Pr\left(Z_{i}=z_{i}|V=\alpha \left(x_{1},x_{2}\right)\right)dx_{2}dx_{1}}. \end{array}$$

The design procedure is given by Algorithm 1. This type of iterative procedure has once been used in other scenarios [34,35]. It is worth noting that the following iterative optimization does not generally guarantee convergence to the global optimum solution. A good choice of initialization can contribute to the avoidance of poor local minima.
**Algorithm 1:** Nonparametric Mapping I**Data**: Initial mapping of $\alpha (x_{1},x_{2})$, the noise for different channels, and δ, which determines the instant at which the iterations will stop.**Result**: Locally optimized $\left(\alpha ,\beta_{1},\beta_{2}\right)$.
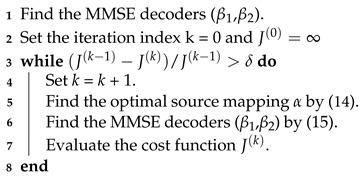


For any given λ, using Algorithm 1 above, we obtain a certain encoder mapping α. The value of λ should be increased if the power $E[\alpha {\left(x_{1},x_{2}\right)}^{2}]$ exceeds the power constraint *P*, and vice versa.

For the actual implementation of (14) and (15), we implemented the following modifications and approximations owing to the fact that it is impossible to evaluate the formulas in the real domain. We generate Monte-Carlo samples from the distribution of X, which is denoted as the set X. We discretize the channel input by a set Y with finite modulation points. The maximum/minimum values of set Y are denoted as $\pm d\frac{L-1}{2}$, where *L* determines the number of points in the set, and *d* denotes the resolution. As the resolution d becomes smaller and the value L becomes larger, the set Y becomes closer to the analog.

The discretized version of (14) is given by

(16) $$\begin{array}{cc} \alpha (x_{1},x_{2})\\ & =\underset{v\in Y}{\arg\min}\left\{Pr\left(z_{1}|v\right)\;\times \;\frac{{\left(x_{1}-\beta_{1}\left(z_{1}\right)\right)}^{2}}{2}+Pr\left(z_{2}|v\right)\;\times \;\frac{{\left(x_{2}-\beta_{2}\left(z_{2}\right)\right)}^{2}}{2}+\lambda \;{\left\|\;v\;\right\|}^{2}\right\}. \end{array}$$

The discretized version of (15) can be expressed as

(17) $$\begin{array}{cc} {\hat{x}}_{1} & =\beta_{1}\left(z_{1}\right)=\frac{\sum_{x_{1}\in X}x_{1}\sum_{x_{2}\in X_{2|x_{1}}}Pr\left(x_{2}|x_{1}\right)Pr\left(z_{1}|\alpha \left(x_{1},x_{2}\right)\right)}{\sum_{x_{1}\in X}\sum_{x_{2}\in X_{2|x_{1}}}Pr\left(x_{2}|x_{1}\right)Pr\left(z_{1}|\alpha \left(x_{1},x_{2}\right)\right)}, \end{array}$$

and

(18) $$\begin{array}{cc} {\hat{x}}_{2} & =\beta_{2}\left(z_{2}\right)=\frac{\sum_{x_{1}\in X}\sum_{x_{2}\in X_{2|x_{1}}}x_{2}Pr\left(x_{2}|x_{1}\right)Pr\left(z_{2}|\alpha \left(x_{1},x_{2}\right)\right)}{\sum_{x_{1}\in X}\sum_{x_{2}\in X_{2|x_{1}}}Pr\left(x_{2}|x_{1}\right)Pr\left(z_{2}|\alpha \left(x_{1},x_{2}\right)\right)}. \end{array}$$

**Figure 2:**
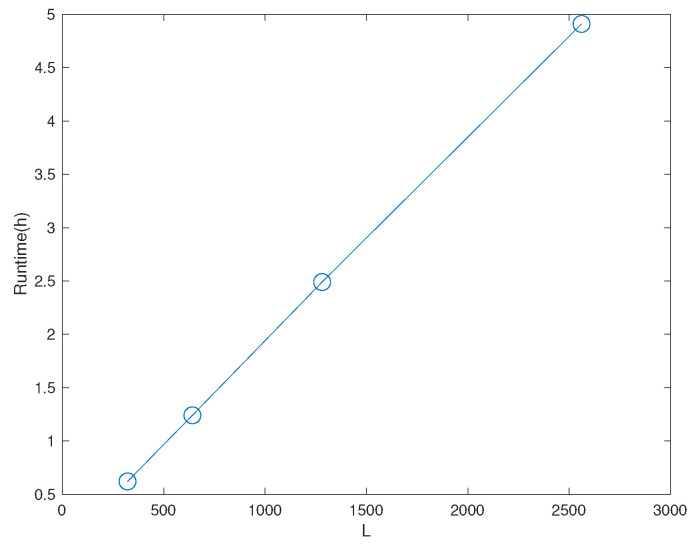
The tradeoff between the value of *L* and the computational cost.

In our experiment, we use $10^{4}$ samples to define the set X. We have also used $\delta =10^{-3}$, and kept d(L−1)/2=4 based on the considerations of the power constraint at the transmitter. The value *L* above is chosen depending on the noise variance, with [1281, 2561] in our experiment, by taking into account the tradeoff between accuracy and computational cost. Figure 2 shows the tradeoff between the value of *L* and the computational cost. The *y* axis shows the runtime to obtain the result for one point in Figure 8b when $\rho ,\sigma_{n_{1}}^{2},\sigma_{n_{2}}^{2}$ are fixed. Its unit is hours.

### 4.2. Nonparametric Mapping II

In the following subsection, we study the functional properties of the unconstrained problem. We obtain an implicit equation for the optimal encoder mapping. Subsequently, we derive the optimal mappings with the necessary conditions above via gradient descent search.

Our system model is symmetrical to some extent, with respect to the nature of the one-bit ADC output and the probability density distributions of the source and noise. We derive below the optimal decoder with the ADC output being 0 and 1 for channel 1, respectively.

(19a) $$\begin{array}{cc} {\hat{X}}_{1}^{0} & =E[X_{1}|Z_{1}=0]\\ \; & =\frac{\int \int x_{1}p\left(x_{1},x_{2}\right)Pr\left(Z_{1}=0|V=\alpha \left(x_{1},x_{2}\right)\right)dx_{2}dx_{1}}{Pr\left(Z_{1}=0\right)} \end{array}$$

(19b) $$\begin{array}{cc} \; & =\frac{\int \int x_{1}p\left(x_{1},x_{2}\right)\left[1-Q\left(\frac{\alpha (x_{1},x_{2})}{\sigma_{n_{1}}}\right)\right]dx_{2}dx_{1}}{Pr(Z_{1}=0)}\\ \; & =\frac{-\int \int x_{1}p\left(x_{1},x_{2}\right)Q\left(\frac{\alpha (x_{1},x_{2})}{\sigma_{n_{1}}}\right)dx_{2}dx_{1}}{Pr(Z_{1}=0)}, \end{array}$$

We elaborate (19a) in detail as follows. While $Z_{1}=0$, it means that $Y_{1}\geq 0$. Hence $N_{1}\geq -\alpha \left(x_{1},x_{2}\right)$. Then we have

(20) $$\begin{array}{cc} Pr(Z_{1}=0|\alpha \left(x_{1},x_{2}\right)) & =Pr(N_{1}\geq -\alpha \left(x_{1},x_{2}\right))\\ \; & =\frac{1}{\sqrt{2\pi }\sigma_{n_{1}}}\int_{-\alpha (x_{1},x_{2})}^{\infty }e^{-\frac{x^{2}}{2\sigma_{n_{1}}^{2}}}dx\\ \; & =Q\left(\frac{\left(-1\right)\alpha \left(x_{1},x_{2}\right)}{\sigma_{n_{1}}}\right)\\ \; & =1-Q\left(\frac{\alpha (x_{1},x_{2})}{\sigma_{n_{1}}}\right). \end{array}$$

In the same way, we could obtain ${\hat{X}}_{1}^{1}$ as

(21) $$\begin{array}{cc} {\hat{X}}_{1}^{1} & =E[X_{1}|Z_{1}=1]\\ \; & =\frac{\int \int x_{1}p\left(x_{1},x_{2}\right)Pr\left(Z_{1}=1|V=\alpha \left(x_{1},x_{2}\right)\right)dx_{2}dx_{1}}{Pr(Z_{1}=1)}\\ \; & =\frac{\int \int x_{1}p\left(x_{1},x_{2}\right)Q\left(\frac{\alpha (x_{1},x_{2})}{\sigma_{n_{1}}}\right)dx_{2}dx_{1}}{Pr(Z_{1}=1)}. \end{array}$$

Based on the results above, we can derive the following relationship,

(22) $$\begin{array}{cc} {\hat{X}}_{1}^{0} & =-{\hat{X}}_{1}^{1}, \end{array}$$

(23) $$\begin{array}{cc} {\hat{X}}_{2}^{0} & =-{\hat{X}}_{2}^{1}. \end{array}$$

Herein, ${\hat{X}}_{j}^{i}$ denotes the estimation when the ADC output is *i* for channel *j*, where i={0,1} and j={1,2}.

The overall average distortion is given by

$$\begin{array}{cc} \bar{D} & =\frac{1}{2}\left(E\left[{\left(X_{1}-{\hat{X}}_{1}\right)}^{2}\right]+E\left[{\left(X_{2}-{\hat{X}}_{2}\right)}^{2}\right]\right)\\ \; & =\frac{1}{2}\left(E\left[\left(X_{1}-{\hat{X}}_{1}\right){\tilde{X}}_{1}\right]+E\left[\left(X_{2}-{\hat{X}}_{2}\right){\tilde{X}}_{2}\right]\right)\\ \; & =\frac{1}{2}\left(E\left[X_{1}{\tilde{X}}_{1}\right]+E\left[X_{2}{\tilde{X}}_{2}\right]\right)\\ \; & =\frac{1}{2}\left(E\left[X_{1}^{2}-X_{1}{\hat{X}}_{1}\right]+E\left[X_{2}^{2}-{\hat{X}}_{2}\right]\right)\\ \; & =\frac{1}{2}\left(\sigma_{X_{1}}^{2}+\sigma_{X_{2}}^{2}-E\left[X_{1}{\hat{X}}_{1}\right]-E\left[X_{2}{\hat{X}}_{2}\right]\right)\\ \; & <\frac{1}{2}\left(\sigma_{X_{1}}^{2}+\sigma_{X_{2}}^{2}\right). \end{array}$$

where (24a) is attributed to the orthogonality of the MMSE estimation. $X_{i}$ denotes the source samples, while ${\hat{X}}_{i}$ and ${\tilde{X}}_{i}$ denote the estimation, and the difference between the source and estimation, respectively. To be more specific, ${\tilde{X}}_{i}=X_{i}-{\hat{X}}_{i}$.

Note that under the MSE distortion criterion, the optimal decoder is the MMSE estimator. The estimation of source $X_{i}$, for example, ${\hat{X}}_{1}$ is obtained as follows,

(25) $$\begin{array}{cc} {\hat{X}}_{1} & =\beta_{1}\left(z_{1}\right)=E\left[X_{1}|Z_{1}=z_{1}\right]=\int x_{1}Pr\left(X_{1}=x_{1}|Z_{1}=z_{1}\right)dx_{1}\\ \; & =\frac{\int x_{1}Pr\left(Z_{1}=z_{1}|X_{1}=x_{1}\right)p\left(x_{1}\right)dx_{1}}{Pr(Z_{1}=z_{1})}\\ \; & =\frac{\int \int x_{1}p\left(x_{1},x_{2}\right)Pr\left(Z_{1}=z_{1}|X_{1}=x_{1},X_{2}=x_{2}\right)dx_{2}dx_{1}}{\int \int p\left(x_{1},x_{2}\right)Pr\left(Z_{1}=z_{1}|X_{1}=x_{1},X_{2}=x_{2}\right)dx_{2}dx_{1}}\\ \; & =\frac{\int \int x_{1}p\left(x_{1},x_{2}\right)Q\left(\frac{{\left(-1\right)}^{z_{1}+1}\alpha \left(x_{1},x_{2}\right)}{\sigma_{n_{1}}}\right)dx_{2}dx_{1}}{\int \int p\left(x_{1},x_{2}\right)Q\left(\frac{{\left(-1\right)}^{z_{1}+1}\alpha \left(x_{1},x_{2}\right)}{\sigma_{n_{1}}}\right)dx_{2}dx_{1}}. \end{array}$$

where (25) is attributed to the fact that $Pr\left(Z_{1}=0|V=\alpha \left(x_{1},x_{2}\right)\right)=Q\left(\frac{\left(-1\right)\alpha \left(x_{1},x_{2}\right)}{\sigma_{n_{1}}}\right)$ while $Pr\left(Z_{1}=1|V=\alpha \left(x_{1},x_{2}\right)\right)=Q\left(\frac{\alpha \left(x_{1},x_{2}\right)}{\sigma_{n_{1}}}\right)$. See (20).

In a similar way, ${\hat{X}}_{2}$ is obtained as,

(26) $$\begin{array}{cc} {\hat{X}}_{2}=\beta_{2}\left(z_{2}\right) & =\frac{\int \int x_{2}p\left(x_{1},x_{2}\right)Q\left(\frac{{\left(-1\right)}^{z_{2}+1}\alpha \left(x_{1},x_{2}\right)}{\sigma_{n_{2}}}\right)dx_{1}dx_{2}}{\int \int p\left(x_{1},x_{2}\right)Q\left(\frac{{\left(-1\right)}^{z_{2}+1}\alpha \left(x_{1},x_{2}\right)}{\sigma_{n_{2}}}\right)dx_{1}dx_{2}}. \end{array}$$

Herein, $z_{1},z_{2}\in \left\{0,1\right\}$. Furthermore, we also notice that the estimation ${\hat{X}}_{i}$ is constant once $z_{i}$ is determined.

Owing to the orthogonality principle of the MMSE estimation, we can verify that $D_{i}=\sigma_{x_{i}}^{2}-E\left[X_{i}{\hat{X}}_{i}\right]$. According to it, we can rewrite the Lagrangian cost function and drop the constants that are independent of α,   

(27) $$\begin{array}{c} L\left(\alpha \right)=-\frac{1}{2}E\left[X_{1}{\hat{X}}_{1}\right]-\frac{1}{2}E\left[X_{2}{\hat{X}}_{2}\right]+\lambda E[\|\alpha \left(X_{1},X_{2}\right){\|}^{2}]. \end{array}$$

Herein, we would like to reemphasize that we use ϕ(·) to denote the pdf of standard normal distribution and ϕ(·,·) to denote the bivariate normal distribution. Q(·) denotes the complementary cumulative distribution function of the standard normal distribution.

By expanding (27), we proceed with the following process,

$$\begin{array}{c} -\frac{1}{2}E\left[X_{1}{\hat{X}}_{1}\right]-\frac{1}{2}E\left[X_{2}{\hat{X}}_{2}\right]+\lambda E\left[\|\alpha \left(X_{1},X_{2}\right){\|}^{2}\right]\\ =\sum_{i=1}^{2}\frac{-1}{2\sigma_{x_{1}}\sigma_{x_{2}}\sigma_{n_{i}}}(\int \int \int_{-\infty }^{-\alpha \left(x_{1},x_{2}\right)/\sigma_{n_{i}}}x_{i}\beta_{i}\left(1\right)\phi \left(\frac{x_{1}}{\sigma_{x_{1}}},\frac{x_{2}}{\sigma_{x_{2}}}\right)\phi \left(\frac{n_{i}}{\sigma_{n_{i}}}\right)dx_{1}dx_{2}dn_{i}\\ \;+\int \int \int_{-\alpha \left(x_{1},x_{2}\right)/\sigma_{n_{i}}}^{\infty }x_{i}\beta_{i}\left(0\right)\phi \left(\frac{x_{1}}{\sigma_{x_{1}}},\frac{x_{2}}{\sigma_{x_{2}}}\right)\phi \left(\frac{n_{i}}{\sigma_{n_{i}}}\right)dx_{1}dx_{2}dn_{i})\\ \;+\frac{\lambda }{\sigma_{x_{1}}\sigma_{x_{2}}}\;\times \;\;\int \int \;\;\phi \left(\frac{x_{1}}{\sigma_{x_{1}}},\frac{x_{2}}{\sigma_{x_{2}}}\right)\alpha^{2}\left(x_{1},x_{2}\right)dx_{2}dx_{1}\\ =\frac{-1}{2\sigma_{x_{1}}\sigma_{x_{2}}}\;\times \;\;\;\int \int \;\;x_{1}\;\left(\;\beta_{1}\left(1\right)Q\left(\frac{\alpha \left(x_{1},x_{2}\right)}{\sigma_{n_{1}}}\right)\;+\beta_{1}\left(0\right)Q\left(\frac{-\alpha \left(x_{1},x_{2}\right)}{\sigma_{n_{1}}}\right)\;\right)\;\times \phi \left(\;\frac{x_{1}}{\sigma_{x_{1}}},\frac{x_{2}}{\sigma_{x_{2}}}\;\right)dx_{2}dx_{1}\\ \;+\frac{-1}{2\sigma_{x_{1}}\sigma_{x_{2}}}\;\times \;\;\;\int \int \;\;x_{2}\;\left(\;\beta_{2}\left(1\right)Q\left(\frac{\alpha \left(x_{1},x_{2}\right)}{\sigma_{n_{2}}}\right)\;+\beta_{2}\left(0\right)Q\left(\frac{-\alpha \left(x_{1},x_{2}\right)}{\sigma_{n_{2}}}\right)\;\right)\;\times \phi \left(\;\frac{x_{1}}{\sigma_{x_{1}}},\frac{x_{2}}{\sigma_{x_{2}}}\;\right)dx_{2}dx_{1}\\ \;+\frac{\lambda }{\sigma_{x_{1}}\sigma_{x_{2}}}\;\int \int \;\phi \left(\frac{x_{1}}{\sigma_{x_{1}}},\frac{x_{2}}{\sigma_{x_{2}}}\right)\alpha^{2}\left(x_{1},x_{2}\right)dx_{2}dx_{1}\\ =\frac{1}{\sigma_{x_{1}}\sigma_{x_{2}}}\;\times \;\;\int \int \;\;[-\frac{x_{1}}{2}\left(\beta_{1}\left(1\right)Q\left(\frac{\alpha \left(x_{1},x_{2}\right)}{\sigma_{n_{1}}}\right)+\beta_{1}\left(0\right)Q\left(\frac{-\alpha \left(x_{1},x_{2}\right)}{\sigma_{n_{1}}}\right)\right)\\ \;-\frac{x_{2}}{2}\left(\beta_{2}\left(1\right)Q\left(\frac{\alpha \left(x_{1},x_{2}\right)}{\sigma_{n_{2}}}\right)+\beta_{2}\left(0\right)Q\left(\frac{-\alpha \left(x_{1},x_{2}\right)}{\sigma_{n_{2}}}\right)\right)\\ \;+\lambda \alpha {\left(x_{1},x_{2}\right)}^{2}]\phi \left(\frac{x_{1}}{\sigma_{x_{1}}},\frac{x_{2}}{\sigma_{x_{2}}}\right)dx_{2}dx_{1}. \end{array}$$

Given that ${\hat{X}}_{i}$ is a discrete random variable with two values: $\beta_{i}\left(0\right)$ and $\beta_{i}\left(1\right)$, the first part of (28a) holds since

$$\begin{array}{c} E[X_{i}{\hat{X}}_{i}]\\ \;=\int x_{i}\beta_{i}\left(0\right)Pr\left({\hat{X}}_{i}=\beta_{i}\left(0\right)|X_{i}=x_{i}\right)p\left(x_{i}\right)dx_{i}\\ \;\;+\;\int x_{i}\beta_{i}\left(1\right)Pr\left({\hat{X}}_{i}=\beta_{i}\left(1\right)|X_{i}=x_{i}\right)p\left(x_{i}\right)dx_{i}\\ \;=\int x_{i}\beta_{i}\left(0\right)Pr\left(Z_{i}=0|X_{i}=x_{i}\right)p\left(x_{i}\right)dx_{i}+\int x_{i}\beta_{i}\left(1\right)Pr\left(Z_{i}=1|X_{i}=x_{i}\right)p\left(x_{i}\right)dx_{i}\\ \;=\frac{1}{\sigma_{x_{1}}\sigma_{x_{2}}\sigma_{n_{1}}}\int \int \int_{-\alpha \left(x_{1},x_{2}\right)/\sigma_{n_{i}}}^{\infty }x_{i}\beta_{i}\left(0\right)\phi \left(\frac{x_{1}}{\sigma_{x_{1}}},\frac{x_{2}}{\sigma_{x_{2}}}\right)\phi \left(\frac{n_{i}}{\sigma_{n_{i}}}\right)dx_{1}dx_{2}dn_{i}\\ \;\;+\;\int \int \int_{-\infty }^{-\alpha \left(x_{1},x_{2}\right)/\sigma_{n_{i}}}x_{i}\beta_{i}\left(1\right)\phi \left(\frac{x_{1}}{\sigma_{x_{1}}},\frac{x_{2}}{\sigma_{x_{2}}}\right)\phi \left(\frac{n_{i}}{\sigma_{n_{i}}}\right)dx_{1}dx_{2}dn_{i} \end{array}$$

where $i^{c}=\left\{1,2\right\}\setminus i$.

Equation (28b) holds due to the following fact that

$$\begin{array}{c} \frac{1}{\sigma_{n_{i}}}\int_{-\infty }^{-\alpha \left(x_{1},x_{2}\right)/\sigma_{n_{i}}}\beta_{i}\left(1\right)\phi \left(\frac{n_{i}}{\sigma_{n_{i}}}\right)dn_{i}+\frac{1}{\sigma_{n_{i}}}\int_{-\alpha \left(x_{1},x_{2}\right)/\sigma_{n_{i}}}^{-\infty }\beta_{i}\left(0\right)\phi \left(\frac{n_{i}}{\sigma_{n_{i}}}\right)dn_{i}\\ =\beta_{i}\left(1\right)Q\left(\frac{\alpha \left(x_{1},x_{2}\right)}{\sigma_{n_{i}}}\right)+\beta_{i}\left(0\right)Q\left(\frac{-\alpha \left(x_{1},x_{2}\right)}{\sigma_{n_{i}}}\right). \end{array}$$

Define $F(\alpha \left(x_{1},x_{2}\right),x_{1},x_{2})$ as:

(29) $$\begin{array}{c} F\left(\alpha \left(x_{1},x_{2}\right),x_{1},x_{2}\right)\\ =\frac{1}{\sigma_{x_{1}}\sigma_{x_{2}}}(-\frac{x_{1}}{2}\left(\beta_{1}\left(1\right)Q\left(\frac{\alpha (x_{1},x_{2})}{\sigma_{n_{1}}}\right)+\beta_{1}\left(0\right)Q\left(\frac{-\alpha (x_{1},x_{2})}{\sigma_{n_{1}}}\right)\right)\\ \;\;\;\;\;\;-\;\frac{x_{2}}{2}\left(\beta_{2}\left(1\right)\cdot Q\left(\frac{\alpha (x_{1},x_{2})}{\sigma_{n_{2}}}\right)+\beta_{2}\left(0\right)\cdot Q\left(\frac{-\alpha (x_{1},x_{2})}{\sigma_{n_{2}}}\right)\right)+\lambda \alpha^{2}\left(x_{1},x_{2}\right)). \end{array}$$

Then after putting $F(\alpha \left(x_{1},x_{2}\right),x_{1},x_{2})$ into (28c), we can apply the necessary condition as below,

(30) $$\begin{array}{cc} \min_{\alpha }\;L\left(\alpha \right) & ≜\;\int \int \;F\left(\alpha \left(x_{1},x_{2}\right),x_{1},x_{2}\right)\phi \left(\frac{x_{1}}{\sigma_{x_{1}}},\frac{x_{2}}{\sigma_{x_{2}}}\right)dx_{2}dx_{1}. \end{array}$$

According to the conclusion in Section 7.5 of [36], when the partial derivative of the function *F* with respect to α, denoted by $F^{\alpha }\left(\alpha \left(x_{1},x_{2}\right),x_{1},x_{2}\right)$ is equal to 0, the function L(α) reaches the minimum. The partial derivative of the function *F* with respect to α is obtained as follows,

(31) $$\begin{array}{c} F^{\alpha }\left(\alpha \left(x_{1},x_{2}\right),x_{1},x_{2}\right)\\ =\frac{1}{\sigma_{x_{1}}\sigma_{x_{2}}}\times (-\frac{x_{1}}{2}\left(\beta_{1}\left(1\right)\left(-\frac{1}{\sqrt{2\pi }\sigma_{n_{1}}}\right)+\beta_{1}\left(0\right)\left(\frac{1}{\sqrt{2\pi }\sigma_{n_{1}}}\right)\right)e^{-\frac{\alpha {\left(x_{1},x_{2}\right)}^{2}}{2\sigma_{n_{1}}^{2}}}\\ \;\;-\;\frac{x_{2}}{2}\left(\beta_{2}\left(1\right)\left(-\frac{1}{\sqrt{2\pi }\sigma_{n_{2}}}\right)+\beta_{2}\left(0\right)\left(\frac{1}{\sqrt{2\pi }\sigma_{n_{2}}}\right)\right)e^{-\frac{\alpha {\left(x_{1},x_{2}\right)}^{2}}{2\sigma_{n_{2}}^{2}}}+2\lambda \alpha \left(x_{1},x_{2}\right)). \end{array}$$

After the deformation of (31), the optimal encoder mapping α subject to the MSE distortion criterion must satisfy the implicit equation as below,

(32) $$\begin{array}{c} 4\sqrt{2\pi }\lambda \alpha \left(x_{1},x_{2}\right)=\frac{x_{1}}{\sigma_{n_{1}}}e^{-\frac{\alpha {\left(x_{1},x_{2}\right)}^{2}}{2\sigma_{n_{1}}^{2}}}\left(\beta_{1}\left(0\right)-\beta_{1}\left(1\right)\right)+\frac{x_{2}}{\sigma_{n_{2}}}e^{-\frac{\alpha {\left(x_{1},x_{2}\right)}^{2}}{2\sigma_{n_{2}}^{2}}}\left(\beta_{2}\left(0\right)-\beta_{2}\left(1\right)\right). \end{array}$$

To find the optimal encoder mapping, we perform the steepest descent search in the opposite direction of the functional derivative of the Lagrangian with respect to the encoder mapping $\alpha (x_{1},x_{2})$ as,

(33) $$\begin{array}{c} \alpha_{i+1}\left(x_{1},x_{2}\right)=\alpha_{i}\left(x_{1},x_{2}\right)-\mu \nabla_{\alpha }L\left(\alpha_{i}\left(x_{1},x_{2}\right)\right), \end{array}$$

where *i* is the iteration index, and μ>0 is the step size.

Hereafter, the gradient of the Lagrangian function L(α) over α is denoted as

(34) $$\begin{array}{ccc} \nabla_{\alpha }L & = & 4\sqrt{2\pi }\lambda \alpha \left(x_{1},x_{2}\right)-\frac{x_{1}}{\sigma_{n_{1}}}e^{-\frac{\alpha^{2}\left(x_{1},x_{2}\right)}{2\sigma_{n_{1}}^{2}}}\left(\beta_{1}\left(0\right)-\beta_{1}\left(1\right)\right)\\ & & -\;\frac{x_{2}}{\sigma_{n_{2}}}e^{-\frac{\alpha^{2}\left(x_{1},x_{2}\right)}{2\sigma_{n_{2}}^{2}}}\left(\beta_{2}\left(0\right)-\beta_{2}\left(1\right)\right). \end{array}$$

The overall design procedure for gradient descent search is given by Algorithm 2.
**Algorithm 2:** Nonparametric Mapping II**Data**: Initial mapping of $\alpha (x_{1},x_{2})$, and the noise for different channels.**Result**: Locally optimized $\left(\alpha ,\beta_{1},\beta_{2}\right)$.
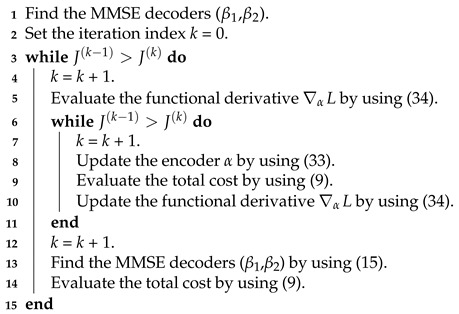


## 5. Parametric Mappings

Compared with the nonparametric mappings, parametric mappings have obvious advantages in terms of their lower computational cost and fixed functional structures. Moreover, they could be updated according to the variations of the signal properties and channel conditions by adjusting their parameters.

**Figure 3:**
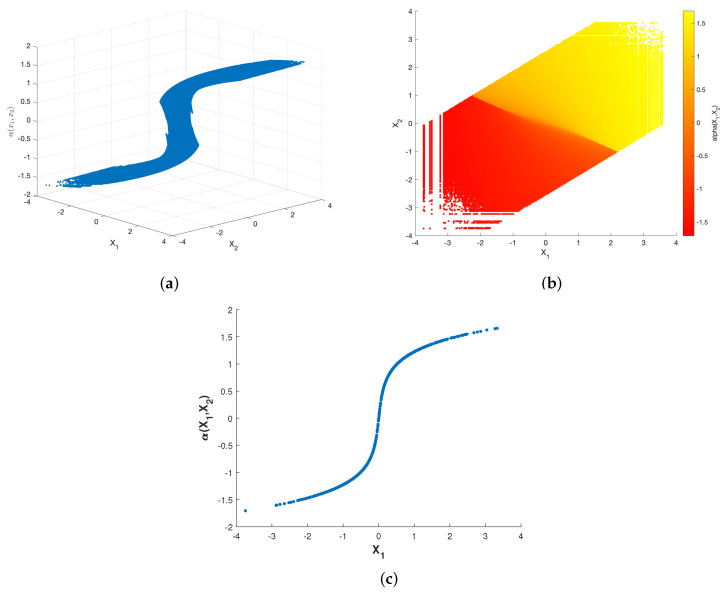
Optimized encoder mapping with Algorithm 1 for $\sigma_{n_{1}}^{2}=0.32$, $\sigma_{n_{2}}^{2}=1$, P=1 and ρ=0.7. The (**a**) shows the curved surface while the (**b**) shows the corresponding two-dimensional representation. The (**c**) shows the curve while $X_{1}=X_{2}$.

**Figure 4:**
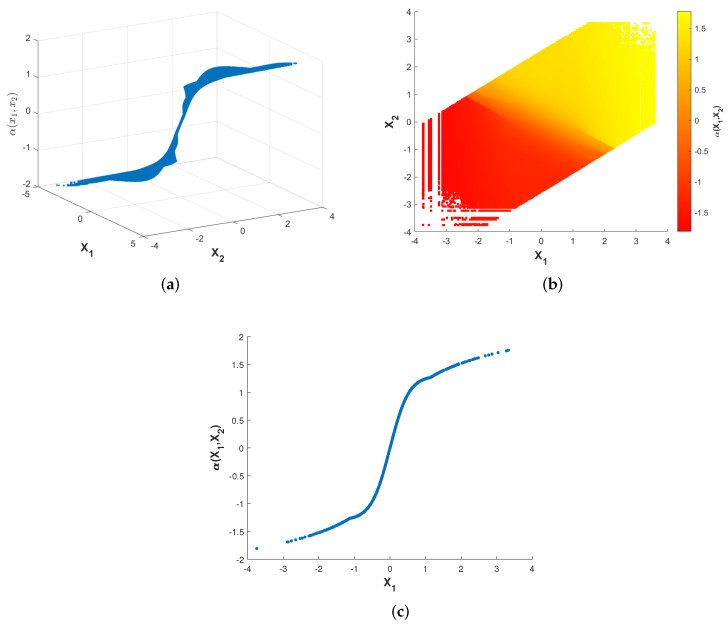
Optimized encoder mapping with algorithm 2 for $\sigma_{n_{1}}^{2}=0.32$, $\sigma_{n_{2}}^{2}=1$, P=1 and ρ=0.7. The (**a**) shows the curved surface while the (**b**) shows the corresponding two-dimensional representation. The (**c**) shows the curve while $X_{1}=X_{2}$.

Figure 3 and Figure 4 show plots of the optimized encoder mapping with Algorithms 1 and 2, respectively. Herein and in the sequel, we define the CSNR as

(35) $$\begin{array}{c} \mathrm{CSNR}=10\log_{10}\left(\frac{NP}{\sum_{i=1}^{N}\sigma_{n_{i}}^{2}}\right). \end{array}$$

Although the structures of the two nonparametric mappings are not exactly the same, we can still summarize some common characteristics. There exist two flat layers in both nonparametric mappings. Different degrees of deformation can be observed in the middle part of two nonparametric mappings surfaces. While fixing $X_{1}=X_{2}$, the curve of $\alpha (X_{1},X_{2})$ with respect to $X_{1}$ is shown as Figure 3c and Figure 4c. The shapes of the two nonparametric mappings obtained above inspire us to propose several different parametric encoding schemes.

After examining the nonparametric mappings mentioned above for different CSNRs and the correlation coefficient ρ, we also notice that due to the symmetry of the system, if ρ=1 and $\sigma_{n_{1}}=\sigma_{n_{2}}$, the problem studied becomes the point-to-point problem presented in [37]. When it comes to the case of ρ=1, $\sigma_{n_{1}}=\sigma_{n_{2}}$ together with the infinite resolution front end, the problem reduces to the one in [38].

### 5.1. Linear Transmission

In [8], the linear scheme for the transmission of bivariate Gaussian sources over a GBC is proposed. The encoder mapping for the linear transmission is given by

(36) $$\begin{array}{c} V=\sqrt{\frac{P}{\sigma_{X}^{2}\left(\omega^{2}+\zeta^{2}+2\omega \zeta \rho \right)}}\left(\omega X_{1}+\zeta X_{2}\right), \end{array}$$

where ω∈[0,1], and ζ=1−ω.

Closed-form expression of the average distortion $\bar{D}$ of linear transmission is hard to be obtained. We choose to substitute (32) into (15) to obtain $\bar{D}$.

### 5.2. Sigmoid-like Function

From Figure 3 and Figure 4, we can observe that there exists a flat platform in the optimized encoding mapping. This feature is similar to the sigmoid function to some extent. Therefore, we propose to adopt the sigmoid-like function, which is defined as

(37) $$\begin{array}{c} \alpha \left(x_{1},x_{2}\right)=\frac{1}{1+e^{-(a_{1}x_{1}+a_{2}x_{2})}}-\frac{1}{2}, \end{array}$$

where $a_{1}$ and $a_{2}$ jointly control the offset angle of the mapping on the X-Y plane and the extension of the mapping surface.

The optimization step can be achieved by an exhaustive search on the parameter space to jointly determine the optimal values for $a_{1}$ and $a_{2}$. The results are obtained via Monte-Carlo optimization of parameters $a_{1}$ and $a_{2}$ in (37) so that the SDR is maximized.

### 5.3. Sinh-like Function

The parametric sine-like mapping in [26] is proposed to satisfy individual quality of service requirements in Gaussian broadcast channels. We adapt the parametric curve structure in our setting and propose the new mapping as indicated below,

(38) $$\begin{array}{c} c_{b_{1},b_{2}}\left(t\right)=U\Sigma^{1/2}s\left(t\right), \end{array}$$

(39) $$\begin{array}{c} s\left(t\right)={\left[s_{x}\left(t\right)\;s_{y}\left(t\right)\right]}^{T}=\left(\begin{array}{c} t^{2}\sinh\left(b_{1}t\right)\\ b_{2}\sinh\left(b_{1}t\right) \end{array}\right), \end{array}$$

where $U^{H}\Sigma U$ is the eigendecomposition of the covariance matrix, with U the matrix consisting of the eigenvectors as columns, and $\Sigma =\mathrm{diag}\left\{\eta_{1},\eta_{2}\right\},\eta_{1}>\eta_{2}$.

The optimization of $b_{1}$ and $b_{2}$ is achieved by exhaustively searching the parameter space.

### 5.4. Shannon-Kotel’nikov-like Function

Shannon-Kotel’nikov (S-K) mappings are studied in previous works such as [39,40]. For the 2:1 bandwidth reduction scenario, the spiral curve is given by

(40) $$\begin{array}{c} s\left(t\right)=\pm \frac{\Delta }{\pi }\left(\cos\left(\varphi \left(ct\right)\right)i+\sin\left(\varphi \left(ct\right)\right)j\right), \end{array}$$

where

$$\begin{array}{c} \varphi \left(\omega t\right)=\sqrt{\frac{c|t|}{\Delta \eta }}. \end{array}$$

We implement the following modifications to the S-K mapping function so that the pitch of the mapping curve varies. In other words, the (radial) distance between the two spiral arms varies all along instead of keeping constant as in previous works.

(41) $$\begin{array}{c} s\left(t\right)=\pm \frac{t^{2}}{\pi }(\cos(c|t|)i+\sin(c|t|)j). \end{array}$$

The optimization of *c* in (41) is achieved by exhaustively searching the parameter space as well.

**Figure 5:**
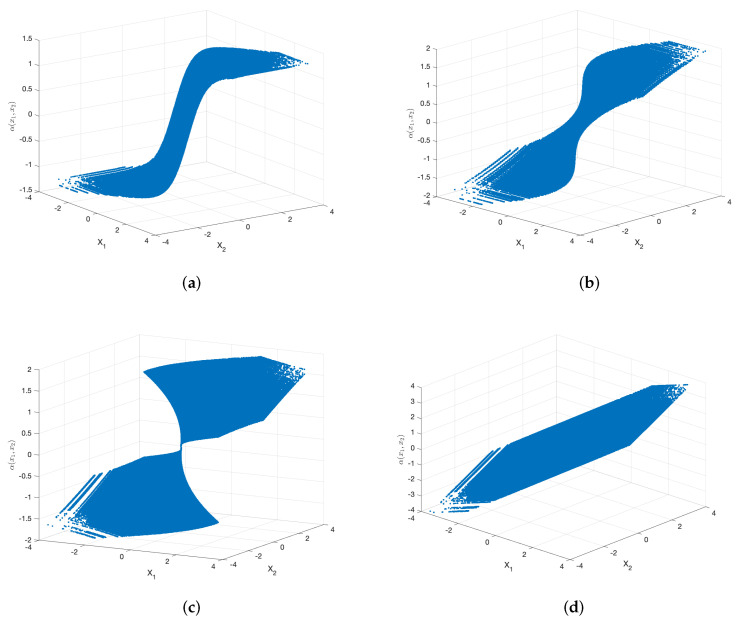
Curved surfaces of the sigmoid-like function, sinh function, S-K-like function and uncoded scheme with optimized parameters for $\sigma_{n_{1}}^{2}=0.32$, $\sigma_{n_{2}}^{2}=1$, P=1 and ρ=0.7. (**a**): sigmoid-like function, (**b**): sinh-function, (**c**): S-K-like function, (**d**): uncoded scheme.

**Figure 6:**
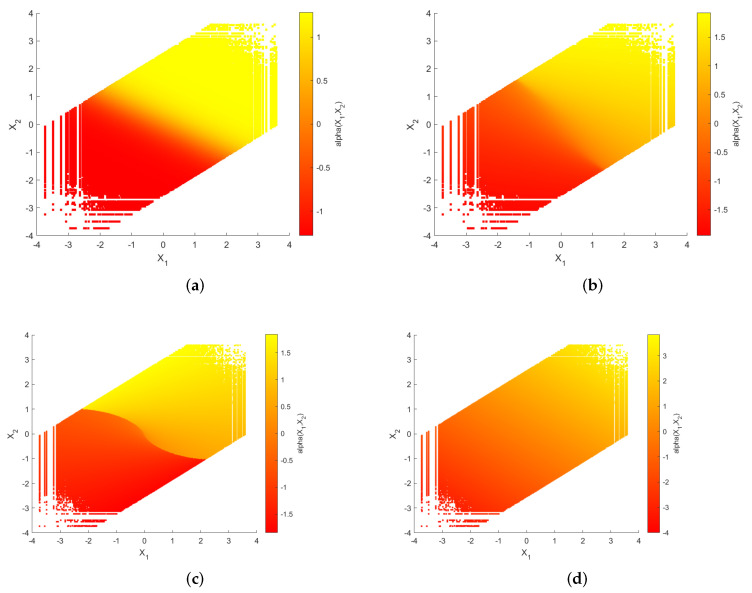
The two-dimensional representations of the curved surfaces of the sigmoid-like function, sinh function, S-K-like function and uncoded scheme with optimized parameters for $\sigma_{n_{1}}^{2}=0.32$, $\sigma_{n_{2}}^{2}=1$, P=1 and ρ=0.7. (**a**): sigmoid-like function, (**b**): sinh-function, (**c**): S-K-like function, (**d**): uncoded scheme.

**Figure 7:**
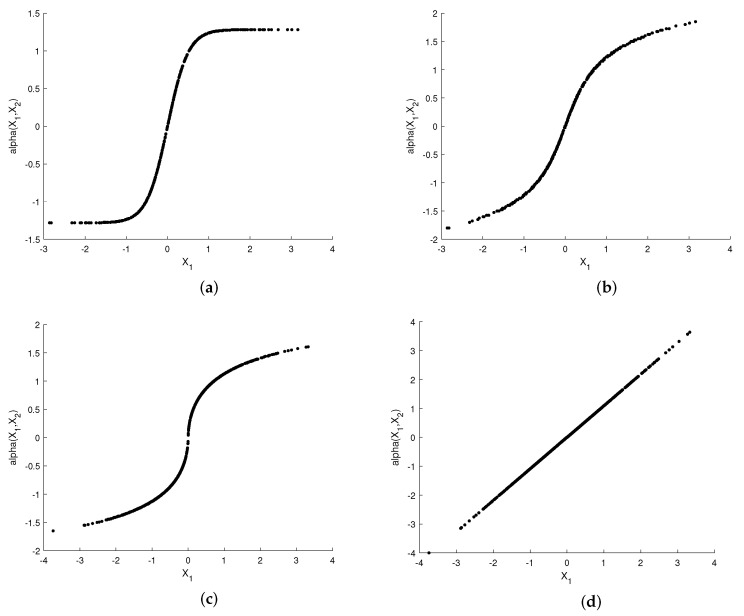
When $X_{1}=X_{2}$, the curves of the sigmoid-like function, sinh function, S-K-like function and uncoded scheme with optimized parameters for $\sigma_{n_{1}}^{2}=0.32$, $\sigma_{n_{2}}^{2}=1$, P=1 and ρ=0.7. (**a**): sigmoid-like function, (**b**): sinh-function, (**c**): S-K-like function, (**d**): uncoded scheme.

The curved surfaces of the sigmoid-like function, sinh function, S-K-like function and uncoded scheme are depicted in Figure 5a–d, respectively. Their corresponding two-dimensional representations are depicted in Figure 6a–d, respectively. While fixing $X_{1}=X_{2},$ the curves of $\alpha (X_{1},X_{2})$ with respect to $X_{1}$ are shown in Figure 7a–d.

## 6. Numerical Results

In this section, we present the performances and validate the effectiveness of the nonparametric and parametric mappings introduced in the previous sections. In the following experiments, the overall MSE is still defined as $\bar{D}=\frac{1}{2}\left(D_{1}+D_{2}\right)$, and signal-to-distortion rate (SDR) is defined as $10\log_{10}\left(\sigma_{X}^{2}/\bar{D}\right)$. The average distortion bound when the infinite resolution ADC front ends are adopted is denoted as bound A, and as bound B when one-bit ADC front ends are adopted.

According to (35), we change the values of CSNR by fixing the channel noise and by varying the values of transmitting power or vice versa. In the following experiments, without loss of generality, $\sigma_{X}$ is set to 1 as in the cases of other values for $\sigma_{X}$, normalization can be adopted.

**Figure 8:**
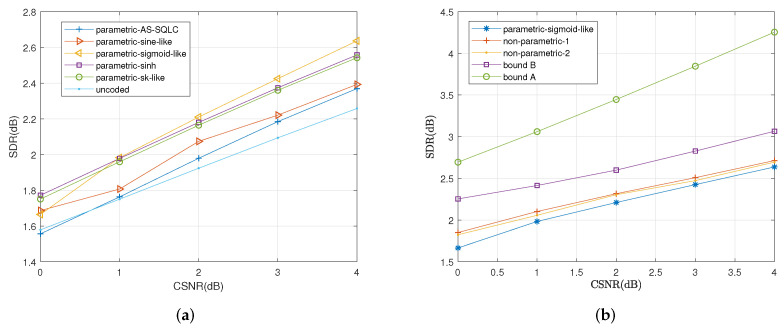
Average distortion performance for $\sigma_{n_{1}}^{2}=0.32$, $\sigma_{n_{2}}^{2}=1$, ρ=0.7 by the relevant schemes at different values of *P*. (**a**): Performance comparisons of all the relevant parametric mappings, (**b**): Performance comparisons of sigmoid-like function, non-parametric mappings and the bounds.

Under the average distortion criterion, we compare the parametric mappings mentioned in Section 5 with two state-of-the-art parametric mappings proposed for the broadcast channel with infinite resolution ADC front ends, the sine-like curve [26] and the alternating sign-scalar quantizer linear coder (AS-SQLC) [28] for different values of CSNR, as shown in Figure 8a. The performance of the sigmoid-like mapping is superior to all the other parametric schemes. Compared with the AS-SQLC scheme and the sine-like scheme, with the exception of the uncoded transmission scheme, the proposed parametric schemes inspired by optimal functional properties yeild better performances.

In Figure 8b, we compare the sigmoid-like function (37) and the two nonparametric mappings with the conditional outer bound under one-bit ADC and the outer bound in the case of an infinite resolution ADC front end. Figure 8b shows the performance curves of the proposed parametric sigmoid-like function and two nonparametric mappings in terms of SDR versus CSNR with correlation coefficient ρ=0.7. Herein, to vary CSNR, the values of $\sigma_{n_{1}}^{2}$ and $\sigma_{n_{2}}^{2}$ in (35) are fixed to 0.56 and 1, respectively, while the average transmitting power is varied. The bound for the scenario with one-bit ADC front end and the bound for the scenario with an infinite resolution front end are indicated in this figure with purple squares and blue circles, respectively.

We observed that with the increase in CSNR, the bound for an infinite resolution front end is increasingly ahead of the bound under the one-bit ADC front end. Two nonparametric mappings outperform the parametric sigmoid-like mappings, where the nonparametric mapping I leads to the nonparametric mapping II. Meanwhile, the performances of two nonparametric mappings approach the bound under one-bit ADC front end.

**Figure 9:**
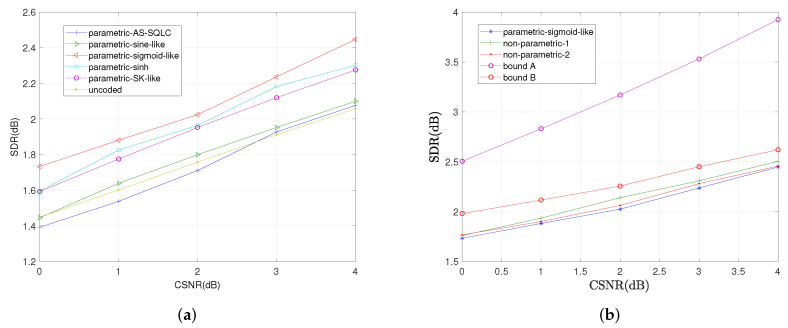
Average distortion performance for $\sigma_{n_{1}}^{2}=0.32$, $\sigma_{n_{2}}^{2}=1$, ρ=0.6 by the relevant schemes at different values of *P*. (**a**): Performance comparisons of all the relevant parametric mappings, (**b**): Performance comparisons of sigmoid-like function, non-parametric mappings and the bounds.

**Figure 10:**
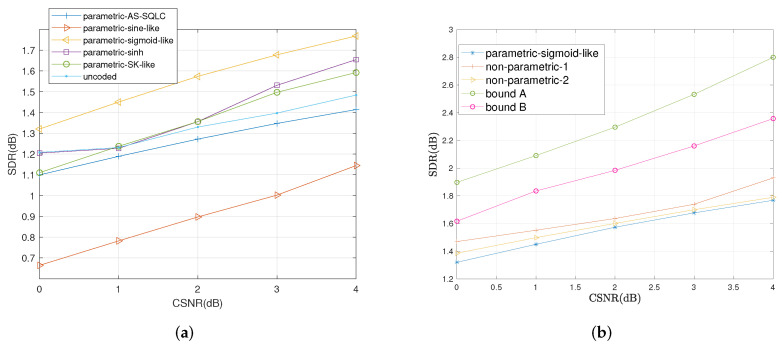
Average distortion performance for $\sigma_{n_{1}}^{2}=0.32$, $\sigma_{n_{2}}^{2}=1$, ρ=0.2 by the relevant schemes at different values of *P*. (**a**): Performance comparisons of all the relevant parametric mappings, (**b**): Performance comparisons of sigmoid-like function, non-parametric mappings and the bounds.

We also compare the average distortions by relevant schemes with the increase in CSNR when ρ=0.6 and ρ=0.2 in Figure 9 and Figure 10, respectively. Similarly, the sigmoid-like mappings perform best within all parametric mappings while nonparametric mapping I performs better than nonparametric mapping II.

**Figure 11:**
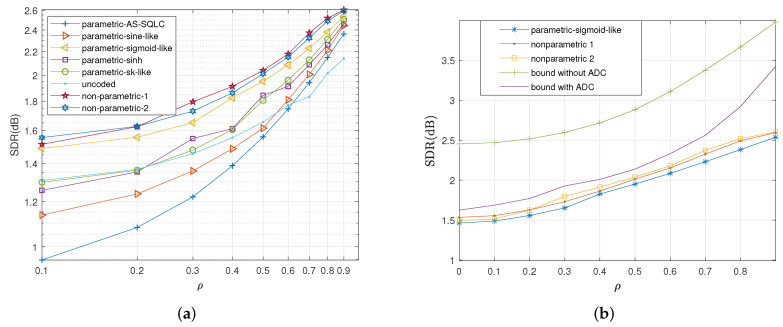
Average distortion performance for $\sigma_{n_{1}}^{2}=0.32$, $\sigma_{n_{2}}^{2}=1$, P=1 with optimized values of parameters at different values of ρ. (**a**): Performance comparisons of all the relevant parametric mappings, (**b**): Performance comparisons of sigmoid-like function, non-parametric mappings and the bounds.

**Figure 12:**
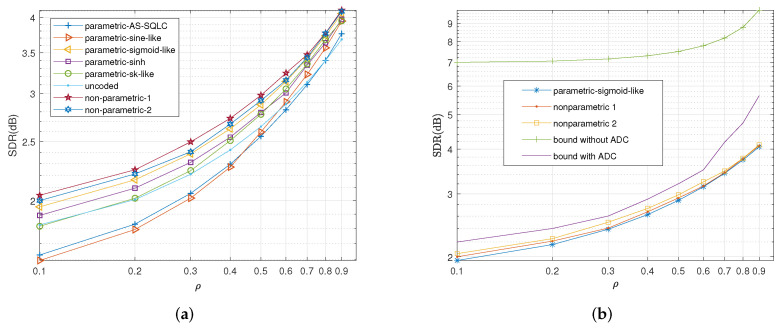
Average distortion performance for $\sigma_{n_{1}}^{2}=0.032$, $\sigma_{n_{2}}^{2}=0.1$, P=1 with optimized values of parameters at different values of ρ. (**a**): Performance comparisons of all the relevant parametric mappings, (**b**): Performance comparisons of sigmoid-like function, non-parametric mappings and the bounds.

In Figure 11 and Figure 12, we plot the SDR versus correlation coefficient ρ when CSNR is equal to 1.8 and 11.8 dB, respectively. Herein, we have kept the transmitting power P=1, while we change the channel noise, with $\sigma_{n_{1}}^{2}=0.32$ and $\sigma_{n_{2}}^{2}=1$ in Figure 11, and $\sigma_{n_{1}}^{2}=0.032$ and $\sigma_{n_{2}}^{2}=0.1$ in Figure 12.

When CSNR is significantly low (e.g., 1.8 dB) as shown in Figure 11, sigmoid-like mapping outperforms all the other parametric mappings at different correlation coefficient values while the sinh mapping and SK-like mapping are both superior to the remaining parametric ones. With the increase in correlation coefficient ρ, the uncoded scheme lags behind the AS-SQLC scheme and the sine-like scheme.

When CSNR increases to 11.8 dB as shown in Figure 12, the sigmoid-like mapping still yields the best performance within all the parametric mappings, and is inferior to the nonparametric ones, while the gap shrinks with the increase in the correlation coefficient ρ. For large values of the coefficient ρ, the performances of the AS-SQLC and sine-like scheme become closer to those of the proposed parametric mappings, while the uncoded scheme gradually lags behind the AS-SQLC and the sine-like schemes.

As observed in the mentioned figures, the proposed sigmoid-like mapping always yields a better performance than the AS-SQLC mapping and sine-like mapping, which are particularly designed for a broadcast channel with infinite resolution ADC front end. When the correlation coefficient ρ decreases, both the gap between the performances of the nonparametric mappings and parametric ones and the gap between the performances of parametric mappings proposed in this work and the AS-SQLC as well as the sine-like scheme expand.

Note that the nonparametric mapping I has a slight lead in the performance compared to the nonparametric mapping II. This is due to the fact that Algorithm 1 has a higher degree-of-freedom to place points in the channel space than Algorithm 2. The above gain comes at the expense of the computational cost.

As CSNR increases, the parametric sigmoid-like mapping approaches more closely to two nonparametric mappings, indicating less gain from the nonparametric algorithms. We attribute this performance to the fact that as the communication condition improves, the influence of the one-bit ADC front end becomes larger, and becomes harder to be compensated by nonparametric mapping algorithms. In low-CSNR cases, when the influence of channel noise outweighs the impact of the one-bit ADC front end, the performance promotions of the two nonparametric mapping algorithms become more obvious.

**Figure 13:**
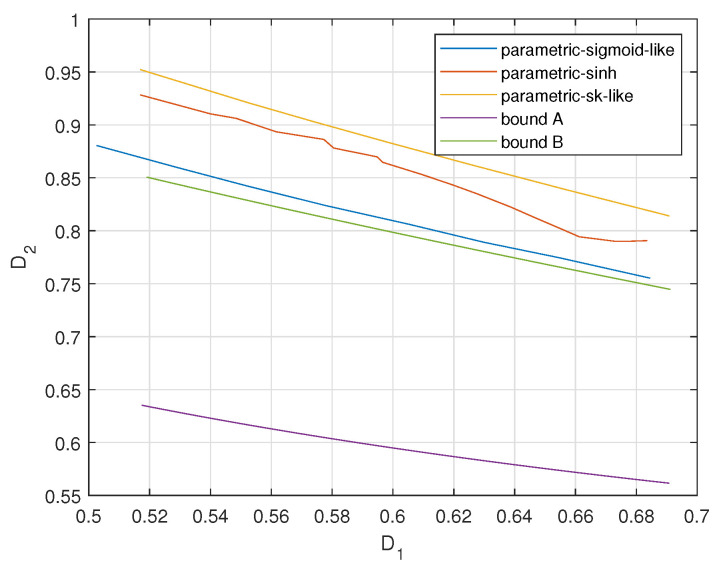
Distortion regions $\left(D_{1},D_{2}\right)$ for $\sigma_{n_{1}}^{2}=0.32$, $\sigma_{n_{2}}^{2}=1$, P=1, ρ=0.2 for three parametric mappings and two bounds.

**Figure 14:**
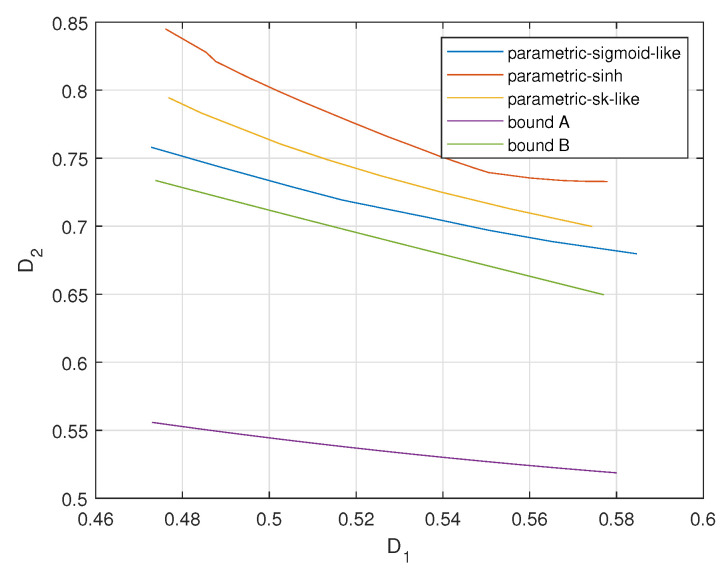
Distortion regions $\left(D_{1},D_{2}\right)$ for $\sigma_{n_{1}}^{2}=0.32$, $\sigma_{n_{2}}^{2}=1$, P=1, ρ=0.6 for three parametric mappings and two bounds.

**Figure 15:**
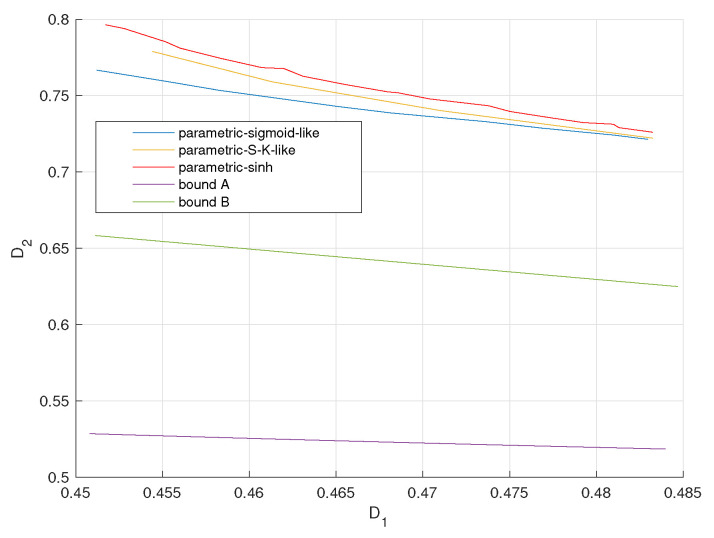
Distortion regions $\left(D_{1},D_{2}\right)$ for $\sigma_{n_{1}}^{2}=0.32$, $\sigma_{n_{2}}^{2}=1$, P=1, ρ=0.7 for three parametric mappings and two bounds.

Figure 13, Figure 14 and Figure 15 plot the achievable distortion bounds for three parametric mappings, the bound with infinite resolution ADC front ends and the conditional outer bound with one-bit ADC front ends when different values are assigned to ρ. We would like to emphasize that the bounds we discuss here are not average distortion bounds in previous figures. These bounds are obtained by searching for minimal attainable $D_{2}$ for given $D_{1}$, as shown in (7). They characterize the attainable distortion regions. To plot the $D_{1}$-$D_{2}$ curves for the proposed parametric encoders in Figure 13, Figure 14 and Figure 15, we varied the parameters to obtain a database for $D_{1}$-$D_{2}$ pairs. Then for a given value of $D_{1}$, we document the corresponding minimal value of $D_{2}$. (It is hard to keep $D_{1}$ constant in the practical experiments. We obtain the values of $D_{1}$ around the given one and document the corresponding $D_{2}$s. Then the minimum $D_{2}$ is found within these $D_{2}$s.) Finally, we could plot the complete $D_{1}$-$D_{2}$ curves for the proposed parametric encoders.

We observe that the bound for an infinite resolution ADC front end is relatively closer to the bound for a one-bit ADC front end with larger ρ values. The sigmoid-like mapping outperforms the other two parametric mappings and is close to the bound for a one-bit ADC front end when ρ is relatively lower.

**Figure 16:**
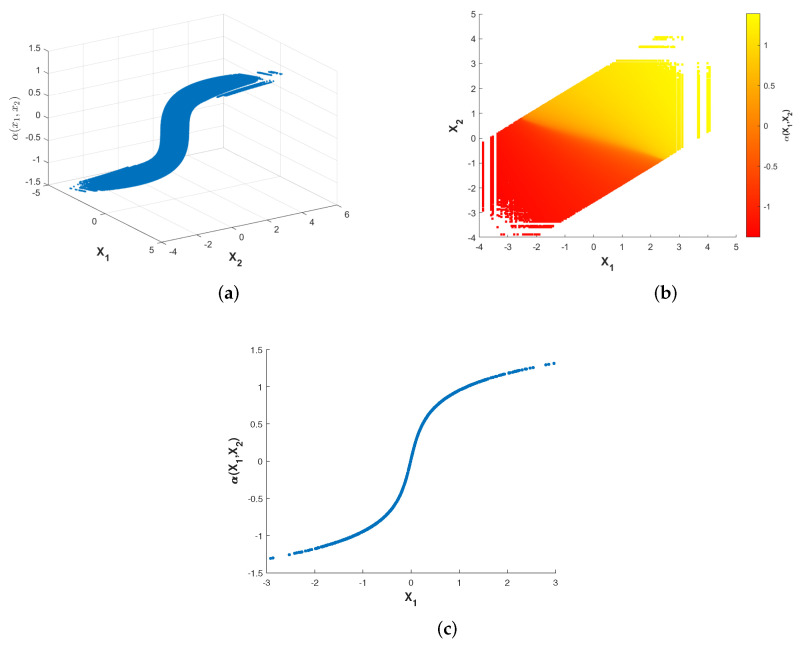
Optimized encoder nonparametric mapping I with CSNR = 0 dB and ρ=0.7. (**a**) shows the curved surface, while the (**b**) shows the corresponding two-dimensional representation. The (**c**) shows the curve while $X_{1}=X_{2}$.

**Figure 17:**
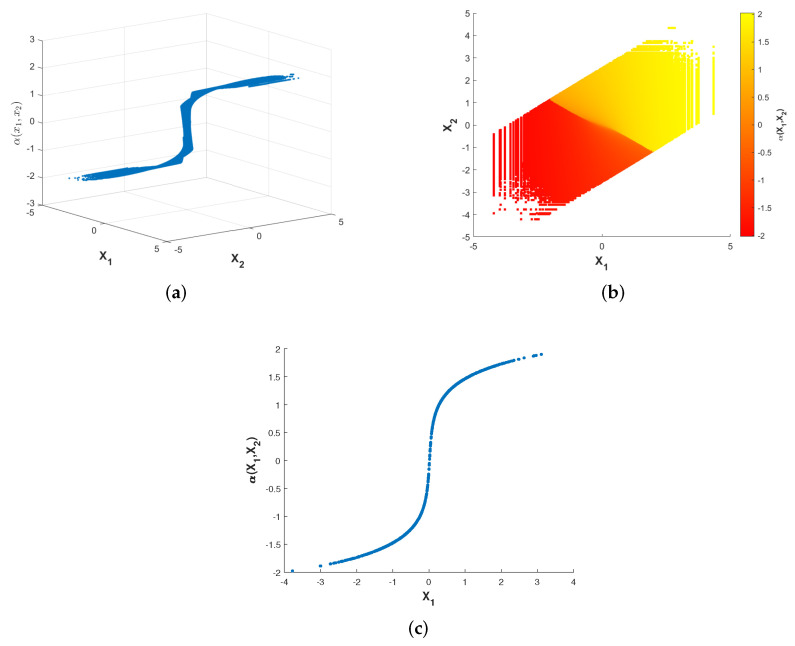
Optimized encoder nonparametric mapping I with CSNR = 4 dB and ρ=0.7. (**a**) shows the curved surface, while the (**b**) shows the corresponding two-dimensional representation. The (**c**) shows the curve while $X_{1}=X_{2}$.

Figure 16 and Figure 17 show the encoder structures of the nonparametric mapping I at two CSNR levels, respectively. As our system model can be approximated as a symmetric one, it is an interesting result that the optimized encoder mappings are odd as well. As CSNR increases, the structure of the encoder mapping is gradually distorted. The deformation above indicates the advantage of the nonparametric mappings compared with the parametric ones, since the former ones have a higher degree-of-freedom for placing points in the channel space rather than being restrained within a fixed structure.

## 7. Conclusions

In this work, we consider the transmission of bivariate Gaussian sources over Gaussian broadcast channels with one-bit ADC front ends. The conditional distortion outer bound for this scenario is derived. Two algorithms are proposed to design the nonparametric mappings. The nonparametric mapping I is achieved based on the iterative optimization between the encoder and the decoder. The nonparametric mapping II is achieved by gradient descent search based on the necessary conditions for the optimal encoder. Based on the characteristics of the optimal encoder mappings, we propose several parametric mappings. Despite a certain extent of performance degradation, the parametric mappings proposed herein can be used in place of the nonparametric mappings as they require lower computational cost and are more adaptable to the channel condition variations. Future extensions of this work include the derivation of the closed-form approximations for the mapping distortion, further design of parametric mappings applied to the system with fading channels, and investigations of the performance of the system with higher level ADC front ends.

## Data Availability

The data presented in this study are available on request from the corresponding author. The data are not publicly available due to the ongoing study in this line of research.

## Appendix A. Proof for Bound under One-Bit ADC Front End

Herein, we derive the genie-aided outer bound while $X_{2}^{n}$ is known at both the transmitter and receiver 1. On the basis of the data processing inequality (DPI), we have

(A1) $$\begin{array}{c} I\left(X_{1}^{n};{\hat{X}}_{1}^{n}|X_{2}^{n}\right)\leq I\left(X_{1}^{n};Z_{1}^{n}|X_{2}^{n}\right). \end{array}$$

The conditional rate-distortion function under the assumption that $X_{2}^{n}$ is known to both encoder and receiver 1, implies the following inequality

(A2) $$\begin{array}{c} I\left(X_{1}^{n};{\hat{X}}_{1}^{n}|X_{2}^{n}\right)\geq \frac{n}{2}\log\frac{\sigma_{X}^{2}\left(1-\rho^{2}\right)}{D_{1|2}^{ADC}}. \end{array}$$

Due to the existence of the Markov chain relationship $\left(X_{1}^{n},X_{2}^{n}\right)↔V^{n}↔Z_{1}^{n}$, mutual information $I(X_{1}^{n};Z_{1}^{n}|X_{2}^{n})$ can be expressed as

(A3) $$\begin{array}{cc} I(X_{1}^{n};Z_{1}^{n}|X_{2}^{n}) & =H\left(Z_{1}^{n}|X_{2}^{n}\right)-H\left(Z_{1}^{n}|X_{1}^{n},X_{2}^{n}\right)\\ & =H\left(Z_{1}^{n}|X_{2}^{n}\right)-H\left(Z_{1}^{n}|V^{n}\right). \end{array}$$

Furthermore, we have the following inequality

(A4) $$\begin{array}{c} H\left(Z_{1}^{n}\right)\geq H\left(Z_{1}^{n}|X_{2}^{n}\right)\geq H\left(Z_{1}^{n}|V^{n}\right). \end{array}$$

As shown in Lemma 2 of [41], since the quantizer is symmetric, it would not lose the optimality to restrict attention to symmetric input distributions. In doing so, as the quantizer and the noise are already symmetric, the probability mass function (PMF) of *Z* is also symmetric. Hence, $H(Z_{1})=1$.

With the one-bit symmetric quantization, the quantized channel output is

(A5) $$\begin{array}{c} Z_{1}^{n}=\Gamma \left(V^{n}+N_{1}^{n}\right). \end{array}$$

Since $Z_{1}$ is binary, it holds that

$$\begin{array}{c} H\left(Z_{1}|V\right)=E\left[-Pr\left(Z_{1}=0|V\right)\log Pr\left(Z_{1}=0|V\right)-Pr\left(Z_{1}=1|V\right)\log Pr\left(Z_{1}=1|V\right)\right], \end{array}$$

then it is easy to derive that

(A6) $$\begin{array}{c} H\left(Z_{1}|V\right)=E\left[h\left(Q\left(\frac{V}{\sigma_{n_{1}}}\right)\right)\right], \end{array}$$

where $Q\left(x\right)=\frac{1}{\sqrt{2\pi }}$$\int_{x}^{\infty }\exp\left(-t^{2}/2\right)dt$, and h(·) denotes the binary entropy function

h(p)=−plog(p)−(1−p)log(1−p),0≤p≤1.

The conclusion is also presented in [41].

Since h(Q(·)) is an even function, we have

$$\begin{array}{c} H\left(Z_{1}|V\right)=E\left[h\left(Q\left(\frac{V}{\sigma_{n_{1}}}\right)\right)\right]=E\left[h\left(Q\left(\frac{\left|V\right|}{\sigma_{n_{1}}}\right)\right)\right]. \end{array}$$

According to [41], the function $h(Q\left(\sqrt{\cdot }\right))$ is a convex function. Jensen’s inequality thus implies

(A7) $$\begin{array}{c} H\left(Z_{1}|V\right)\geq h\left(Q\left(\sqrt{P/\sigma_{n_{1}}^{2}}\right)\right)=h\left(Q\left(\sqrt{{\mathrm{CSNR}}_{1}}\right)\right). \end{array}$$

with equality iff $V^{2}=P$.

On the other hand, $h(Q\left(\sqrt{0\times P/\sigma_{n_{1}}^{2}}\right))=1$, hence there exists γ∈[0,1] such that

$$H\left(Z_{1}|X_{2}\right)=h\left(Q\left(\sqrt{\gamma P/\sigma_{n_{1}}^{2}}\right)\right).$$

Therefore,

(A8) $$\begin{array}{cc} I(X_{1}^{n};{\hat{X}}_{1}^{n}|X_{2}^{n}) & \leq I(X_{1}^{n};Z_{1}^{n}|X_{2}^{n})\\ & =H\left(Z_{1}^{n}|X_{2}^{n}\right)-H\left(Z_{1}^{n}|V^{n}\right)\\ & =\sum_{k=1}^{n}\left[H\left(Z_{1,k}|X_{2}^{n}Z_{1}^{k-1}\right)-H\left(Z_{1,k}|V^{n}Z_{1}^{k-1}\right)\right]\\ & \overset{\left(a\right)}{=}\sum_{k=1}^{n}\left[H\left(Z_{1,k}|X_{2,k}\right)-H\left(Z_{1,k}|V_{k}\right)\right]\\ & \leq n\left(h\left(Q\left(\sqrt{\gamma P/\sigma_{n_{1}}^{2}}\right)\right)-h\left(Q\left(\sqrt{P/\sigma_{n_{1}}^{2}}\right)\right)\right), \end{array}$$

where γ∈[0,1] and (a) follows by the sample-by-sample (zero-delay) encoding assumption. As a result, the following inequality holds

(A9) $$\begin{array}{c} \frac{1}{2}\log\frac{\sigma_{X}^{2}\left(1-\rho^{2}\right)}{D_{1}^{ADC}}\leq \frac{1}{2}\log\frac{\sigma_{X}^{2}\left(1-\rho^{2}\right)}{D_{1|2}^{ADC}}\leq h\left(Q\left(\sqrt{\gamma P/\sigma_{n_{1}}^{2}}\right)\right)-h\left(Q\left(\sqrt{P/\sigma_{n_{1}}^{2}}\right)\right). \end{array}$$

On the other hand, applying the data processing inequality at receiver 2, we obtain the following inequality

(A10) $$\begin{array}{c} \frac{n}{2}\log\frac{\sigma_{X}^{2}}{D_{2}^{ADC}}\leq I\left(X_{2}^{n};{\hat{X}}_{2}^{n}\right)\leq I\left(X_{2}^{n};Z_{2}^{n}\right). \end{array}$$

Additionally, the mutual information can be expressed as

(A11) $$\begin{array}{cc} I(X_{2}^{n};Z_{2}^{n}) & =H\left(Z_{2}^{n}\right)-H\left(Z_{2}^{n}|X_{2}^{n}\right)\\ & =n-nH(Z_{2}|X_{2}). \end{array}$$

Note that

(A12) $$\begin{array}{c} H\left(Z_{2}^{n}|X_{2}^{n}\right)=nh\left(Q\left(\sqrt{\gamma P/\sigma_{n_{2}}^{2}}\right)\right), \end{array}$$

we can thus have

(A13) $$\begin{array}{c} \frac{1}{2}\log\frac{\sigma_{X}^{2}}{D_{2}^{ADC}}\leq 1-h\left(Q\left(\sqrt{\gamma P/\sigma_{n_{2}}^{2}}\right)\right). \end{array}$$

Based on (A9) and (A13), we have

(A14) $$\begin{array}{cc} D_{1}^{ADC} & \geq \frac{\sigma_{X}^{2}\left(1-\rho^{2}\right)}{2^{2\left(h\left(Q\left(\sqrt{\gamma P/\sigma_{n_{1}}^{2}}\right)\right)-h\left(Q\left(\sqrt{P/\sigma_{n_{1}}^{2}}\right)\right)\right)}}, \end{array}$$

(A15) $$\begin{array}{cc} D_{2}^{ADC} & \geq \frac{\sigma_{X}^{2}}{2^{2\left(1-h\left(Q\left(\sqrt{\gamma P/\sigma_{n_{2}}^{2}}\right)\right)\right)}}. \end{array}$$
